# Allergic Contact Dermatitis to 1.6‐Hexanediol Diacrylate in Ski Boots

**DOI:** 10.1111/cod.70005

**Published:** 2025-07-29

**Authors:** Nadia Raison‐Peyron, Alison Poncy, Jakob Dahlin, Cecilia Svedman

**Affiliations:** ^1^ Department of Dermatology Montpellier University Hospital and Montpellier University Montpellier France; ^2^ Department of Occupational and Environmental Dermatology Lund University, Skåne University Hospital Malmö Sweden

**Keywords:** (meth)acrylates, 1.6‐hexanediol diacrylate, allergic contact dermatitis, CAS no. 13048–33‐4, case report, ski boots, sport equipment

We report the case of a 48‐year‐old male patient, a cutler by profession, referred for recurrent eczema on his lower limbs sparing the feet. His medical history included allergic rhinoconjunctivitis to grass, multiple sclerosis, sleep apnoea, and a previous but unspecified skin reaction to adhesive dressings.

On three separate occasions during downhill ski training, the patient developed a similar clinical presentation of erythematous, vesicular, pruritic lesions on his lower legs while wearing a new pair of thermoformed ski boots whose interior part was composed of foam covered with black fabric in contact with legs but not feet (Figure 1A–C). Prior to the episodes with eczema, the patient had worn the boots twice with no reaction.

**FIGURE 1:**
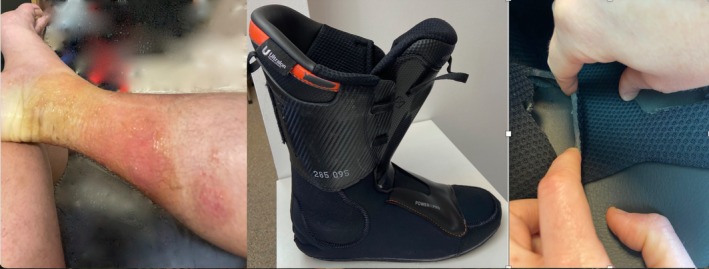
(A) Eczematous rash of the lower limbs sparing feet after wearing ski boots. (B) Ski boots. (C) Grey foam covered with black fabric.

Patch testing was carried out on the patient's back using IQ Ultra Chambers (Chemotechnique Diagnostics, Vellinge, Sweden), occluded for 2 days with Opertape (Iberhospitex, Innovative Health Technologies, Barcelona, Spain), and reading was performed on Day (D)2, D3, and D7 according to ESCD guidelines [1].

The baseline European series (Chemotechnique Diagnostics, Vellinge, Sweden) showed a single positive reaction to 2‐hydroxyethyl methacrylate (2‐HEMA) 2% in petrolatum (pet), ++ on D2 and D3. Ski boot foam scrapings (extracted using water, ethyl alcohol, and acetone) were also all strongly positive (+++) on D2 and D3 (Figure 2A). Conversely, the rubber and plastics/glues series, including acetophenone azine 1% pet, were all negative. Late readings on D7 revealed no further positive results.

**FIGURE 2:**
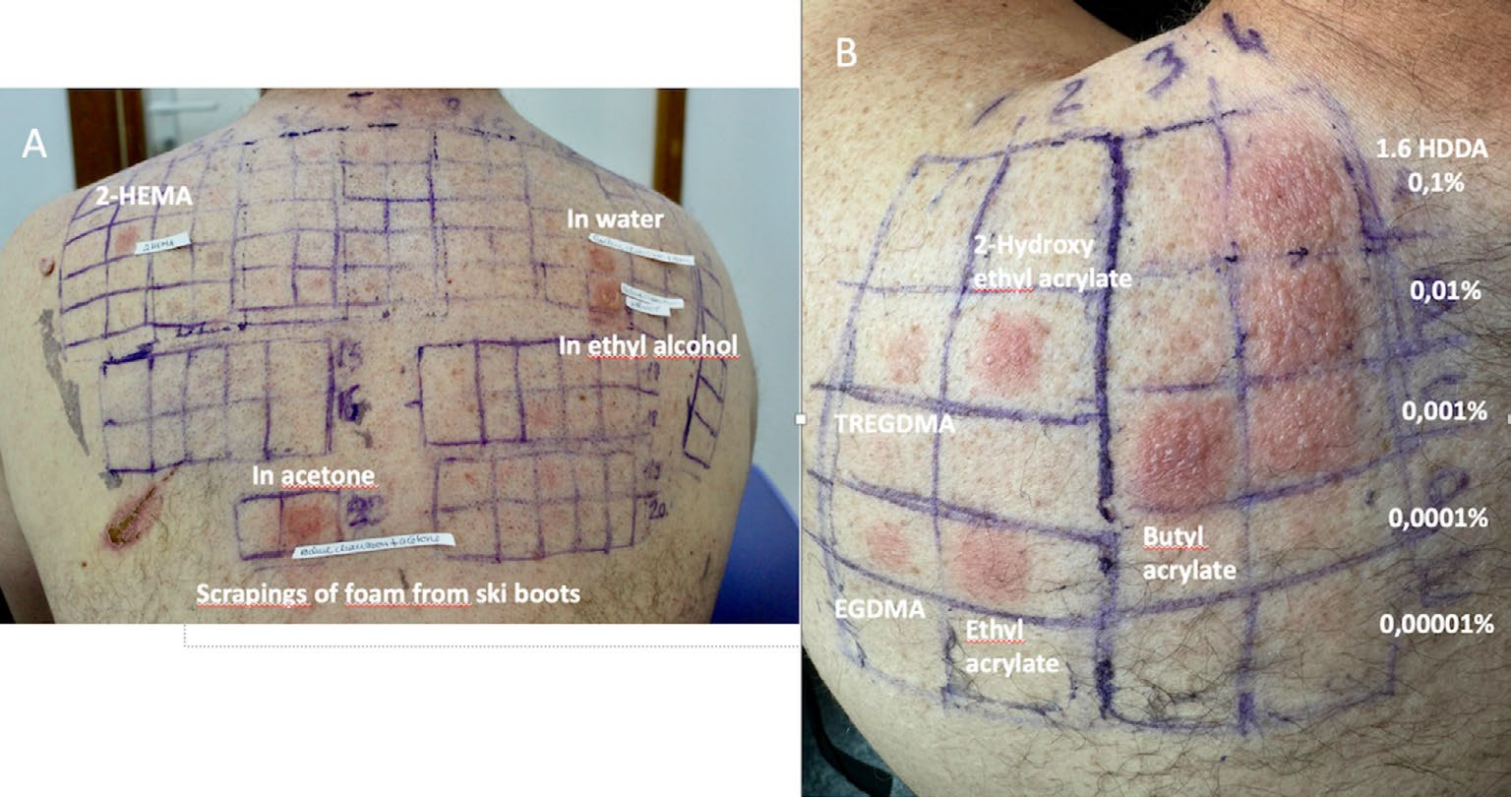
(A) Positive patch tests to 2‐HEMA 2% pet (++) and scrapings of boot liners foam (+++) diluted in water, ethyl alcohol and acetone on D3. (B) Positive patch tests to 1.6 HDDA using serial dilutions at the three highest concentrations 0.1%, 0.01%, and 0.001% pet (+++), butyl acrylate 0,1% pet (+++), ethyl acrylate 0,1% pet (++), 2‐hydroxyethyl acrylate 0,1% pet (++), TREGMA 2% pet (+) and EGDMA 2% pet (+) on D3.

A chemical analysis of the foam sample via gas chromatography–mass spectrometry performed in Sweden revealed a significant quantity of 1,6‐hexanediol diacrylate (1,6 HDDA). The concentration of 1,6‐HDDA in the ski‐boot foam scraping was about 350 ppm (0.035%). Subsequently, we therefore performed additional patch testing with an acrylates series and 1,6 HDDA 0.1% pet, using serial dilutions. 1,6 HDDA showed a strong positive reaction at the three highest concentrations (0.1%, 0.01%, and 0.001%). Other substances tested also elicited positive reactions, including triethylene glycol dimethacrylate (TREGMA) 2% pet, ethylene glycol dimethacrylate 2% pet (EGDMA), 2‐hydroxyethyl acrylate 0.1% pet, ethyl acrylate 0.1% pet, and butyl acrylate 0.1% pet (Figure 2B).

One year later, the patient skied again with the same ski boot shells, but with a different brand of new booties (still thermoformed). The dermatitis quickly relapsed on his lower legs. Then, he continued to ski, wrapping his lower legs in cellophane under socks, without any reaction.

## 1 Discussion

Rubber, biocides, dyes, acetophenone azine, and formaldehyde resins are commonly implicated in allergic contact dermatitis elicited by individual sports equipment [2, 3], while the main sources of acrylate exposure are typically found in nail care (e.g., artificial nails and semi‐permanent varnishes), dentistry (e.g., composite resins), adhesive dressings, glues, and printing inks [4, 5, 6].

2‐HEMA is usually considered a marker for allergy to methacrylates, with a high incidence of cross‐reactions to other methacrylates, which is the reason why it was included in the baseline European series in 2019 [6, 7]. 1,6 HDDA (CAS no. 13048‐33‐4) is a multifunctional acrylate and a potent sensitiser. Several cases of allergic contact dermatitis to 1,6 HDDA have already been reported in the literature, including one related to the coating of a glucose sensor transmitter, another involving medical devices used by patients with ostomy, and the last one linked to a hospital wristband [8, 9, 10]. A case of toxic epidermal necrolysis–like contact dermatitis caused by ultraviolet‐cured inks containing 1,6 HDDA has also been described [11].

The patient had worn the ski boots twice before the dermatitis occurred without any reaction, and when patch tested, was highly positive also at extremely low concentrations of 1,6 HDDA (0.001%) indicating that the sensitisation occurred through the boots. The sensitisation as such is surprising; the possibility of a manufacturing defect of the first ski boots, with excessive quantities of 1,6 HDDA in the glue used to bond the black fabric to the foam, or in the materials themselves that make up the liner might be considered.

The recurrence of the dermatitis with a new pair of boot liners from a different brand suggests the presence of (meth)acrylates, although not identified.

Efforts have been made to investigate how commonly reactions like these are, but we couldn't obtain any information from the ski boot manufacturer or the manufacturer of the foam inside the boot liners.

## Conflicts of Interest

The authors declare no conflicts of interest.

## Data Availability

Data sharing is not applicable to this article as no new data were created or analyzed in this study.
